# Deficiency of Duffy Antigen Receptor for Chemokines Ameliorated Cochlear Damage From Noise Exposure

**DOI:** 10.3389/fnmol.2018.00173

**Published:** 2018-05-30

**Authors:** Bouchra Edderkaoui, Liana Sargsyan, Alisa Hetrick, Hongzhe Li

**Affiliations:** ^1^Research Service, VA Loma Linda Healthcare System, Loma Linda, CA, United States; ^2^Loma Linda University School of Medicine, Loma Linda, CA, United States; ^3^Department of Otolaryngology—Head and Neck Surgery, Loma Linda University School of Medicine, Loma Linda, CA, United States

**Keywords:** Duffy antigen receptors for chemokines, noise exposure, cochlear inflammation, synaptopathy, hearing protection

## Abstract

Cochlear inflammatory response to various environmental insults, including acoustic and ototoxic overexposures, has been increasingly become a topic of interest. As the immune response is associated with both pathology and protection, targeting specific components of the immune response is expected to dissect the relationships between cellular damage and inflammation-associated protection and repair in the cochlea. Duffy antigen receptor for chemokines (DARC) is a member of a group of atypical chemokine receptors, and essential for chemokine-regulated leukocyte/neutrophil trafficking during inflammation. Previous studies have reported that *Darc* deficiency alters chemokine bioavailability and leukocyte homeostasis, leading to significant anti-inflammatory effects in tissues following injury. In this study, we have used *Darc* knockout mice to determine the impact of a deficiency in this gene on cochlear development, as well as function in cochlea subjected to various stresses. We observed that DARC is not required for normal development of cochlear function, as evidenced by typical hearing sensitivity in juvenile *Darc-*KO mice, as compared to wild type (WT) *C57BL/6* mice. However, *Darc-*KO mice exhibited improved hearing recovery after intense noise exposure when compared to wild-type. The auditory brainstem response (ABR) threshold shift between KO and WT mice was most obvious at 1-week post-noise exposure. At cochlear locations above the frequency range of the energy band of damaging noise, both hair cell survival and ribbon synapse density were improved in *Darc* deficient animals. In addition, the mRNA levels of some major inflammatory effectors, including *Mcp-1* and *Gdf15*, were altered in *Darc-*KO mice compared to control mice at 1, 3 and 7 days post-noise exposure. These data collectively suggest that the normal *Darc*-dependent inflammatory response slows down the process of hearing recovery, and exacerbates cellular damage in the cochlea after noise exposure.

## Introduction

Inflammatory responses may occur in all mammalian tissues, organs and systems, including previously recognized “immune privileged” sites such as the knee joint and inner ear. It has been previously thought that noise-induced hearing loss (NIHL) was associated with oxidative stress. However, more recently it has been shown that inflammation also plays a major role in the ear pathology caused by Noise (Tan et al., 2016; Fuentes-Santamaria et al., 2017). Cochlear inflammation that occurs after acoustic or ototoxic drug overexposure is considered sterile inflammation, manifested by elevated cytokine and chemokine levels and infiltrated leukocytes (Tan et al., 2016; Wood and Zuo, 2017). The overall course of the inflammatory response, from its initiation to its resolution, if completed swiftly, is beneficial and protects the host from infection and the risk of further tissue injury (Kalinec et al., 2017). Several anti-inflammatory agents have been investigated as potential therapeutic strategies to curtail extended cochlear inflammation, in order to reduce the risk of hearing loss. These include corticosteroids such as dexamethasone, and nonsteroidal anti-inflammatory drugs such as aspirin (Sha et al., 2006; Behnoud et al., 2009; Piu et al., 2011; Harrop-Jones et al., 2016). Promising results have been achieved using these aggressive anti-inflammatory approaches, but to date none of these drugs have been deemed as hearing loss medicine. Since the inflammatory response is associated with both pathology and protection, we reasoned that targeting a specific genetically predisposed and manipulatable immune component would help to dissect the interactions between the cellular damage and the immunoprotective events in the cochlea.

Duffy antigen receptor for chemokines (DARC) is a transmembrane protein expressed on vascular endothelium in a variety of organs, as well as on red blood cells and certain epithelial cells (Miller et al., 1975; Chaudhuri et al., 1997), cell types that play key role in the processes of inflammation and wound healing (McGrath and Emery, 1985; Bao et al., 2009; Harrison et al., 2011; Karsten et al., 2018). Furthermore, a study by Horuk et al. (1996) has shown that DARC is also expressed in cerebellar Purkinje neurons. DARC binds chemokines of both the C-C and C-X-C families. Our previous studies (Rundle et al., 2013; Alemi et al., 2016) have demonstrated the importance of DARC in inflammation, both in response to bone fracture and bacterial infections. However, its role in post-noise exposure has never been analyzed. Therefore, given the emerging evidence suggesting that inflammation affects hearing loss, our goal in this study was to determine whether DARC is involved in noise induced hearing loss and recovery.

In the present study, we first documented the hearing recovery process after intensive noise exposure in the cochleae of mice with *Darc* deficiency, and evaluated the noise-altered expression level of genes encoding effectors of inflammation. In addition, posttraumatic hair cell survival and ribbon synapse density were also evaluated. Hair cells, primarily outer hair cells (OHCs), as the source of cochlear amplifier are essential for sensitive hearing, and their apical filamentous structure is vulnerable under acoustic challenge (Dallos, 1992, 2008; Li et al., 2015). In contrast, a complete profile of ribbon synapses from the inner hair cell (IHC), is critical in maintaining the temporal cues of acoustic signals. Temporal acuity is necessary for many auditory functions such as sound localization, listening in noise, and spectral segregation (Sinex et al., 2003, 2005; Moser et al., 2006; Moser and Starr, 2016). It is widely recognized that noise exposure results in cochlear synaptopathy, characterized by rapid and reversible synaptic loss, independent of the elevation of hearing threshold (Kujawa and Liberman, 2009, 2015; Liberman and Kujawa, 2017). In terms of the etiology, noise-induced damage to the postsynaptic terminal has been rationalized as being due to excitotoxicity (Pujol and Puel, 1999). However, to date, the mechanism of noise-induced presynaptic ribbon damage remains unknown, while the contribution of cochlear inflammation has only recently been proposed (Kalinec et al., 2017). Here, we assessed cochlear synaptopathy under the DARC-deficiency alleviated inflammatory condition, to gauge the contribution of inflammation onto synaptic ribbon loss.

## Materials and Methods

### Mice

*Darc-knockout* (KO) mice (Dr. Chaudhuri, New York Blood Center, NY, USA) and wild type (WT) control *C57BL/6* (JAX stock #0664) mice were used in this study. Animals were housed in a Specific Pathogen Free-modified room, without sound treatment of ambient noise. All animal work performed in this study was carried out using protocols approved by the Institutional Animal Care and Use Committee of the Jerry L. Pettis VA Medical Center, Loma Linda, CA, USA. Animal use procedures conform with federal regulations regarding personnel, supervision, record keeping and veterinary care.

The *Darc-*KO mice used in this study were initially generated in a mixed *129S1 (129)* and *C57BL/6J (B6)* background as previously described (Luo et al., 2000). The initial *Darc-*KO mice have since been vigorously backcrossed with *B6* mice for at least 10 generations to minimize the genetic contribution of the *129* strain. After 10 backcrosses, the genetic contribution of the donor strain is estimated to be <0.1% (Silver, 1995). *B6* mice are used as the WT control mice in the present study and all auditory phenotypical findings are extremely improbable resulted from the minuscule residue (<0.1%) of *129* genetic background.

### ABR Measurement

Each ear of an anesthetized mouse (ketamine 65 mg/kg and xylazine 13 mg/kg, *i.p*.) from either strain, was stimulated individually with a closed tube sound delivery system sealed into the ear canal. The auditory brainstem responses (ABRs) to tone burst stimuli (5-ms duration, 1-ms rise/fall) at 4, 8, 12, 16, 24 and 32 kHz, with 5 dB steps, was recorded using a TDT System 3 (Tucker-Davis Technologies, Alachua, FL, USA), thresholds determined, and Wave-I peak-to-peak amplitudes measured. ABRs were also recorded before and after noise exposure.

### Noise Exposure

Seven-week-old mice of either sex were used to achieve measurable inflammatory response in the cochlea. Specifically, age-matched *Darc*-KO and WT *B6* control mice were individually caged and exposed to octave-band noise (OBN) for 2 h inside a single-wall acoustic chamber (Industrial Acoustics Company, INC, NY, USA). The cages were situated on a platform, rotated at the rate of 5 min per turn, to ensure a uniform sound level of noise exposure. The sound level was 112 dB SPL and a 1/4″ microphone (Brüel and Kjær, Nærun, Denmark) located proximately above the cage was used to measure the sound level in real time. After noise exposure, the animals were euthanized at different time points and cochleae collected to analyze the effect of intense noise exposure on hair cell survival, neuronal damage, and to evaluate the gene expression changes in response to the noise trauma.

### Extraction of Total Cochlear RNA for Quantitative PCR

Cochleae were rapidly removed, frozen in liquid nitrogen, and then stored at −80°C until further processed. For extraction of total RNA, frozen samples were transferred from cryopreservation tubes to RNase-free sample tubes in dry ice, ground using a RPI micro grinder (Research Product International, Mt. Prospect, IL, USA), and total RNA extracted using the RNeasy Mini Kit (Qiagen, Valencia, CA, USA). Briefly, freshly made 600 μl RLT buffer with 6 μl β-mercaptoethanol was added to each sample then mixed well. Samples were centrifuged for 3 min (Sorvall Legend Mach 1.6R, Thermo Fisher Scientific Inc., Waltham, MA, USA) at 15,000 *g* and supernatants collected into RNase-free tubes and mixed 1:1 with fresh 70% ethanol. Mixtures were then transferred to spin columns in 2 ml collection tubes, centrifuged for 15 s, and the flow through discarded. Columns were washed with RW1 buffer and RPE buffer, with centrifugation, and RNA products were eluted with 40 μl RNase-free water. RNA samples were stored at −80°C and RNA concentrations determined using a spectrophotometer (ND-1000, NanoDrop Technologies, Wilmington, DE, USA).

### Evaluation of mRNA Changes in Response to Noise Exposure

To evaluate the change in mRNA in response to noise exposure, first strand cDNA was generated using 250 ng of total RNA and following the manufacturer’s instructions (Promega, Madison, WI, USA). mRNA level was quantified in the ABI Prism 7900HT Sequence Detection System (Applied Biosystems, Foster City, CA, USA) as recommended by the manufacturer and using SYBR Green (Applied Biosystems) as a detector dye and pre-designed primers (Table 1) for the genes of interest. The data were analyzed using ViiA™ 7 Software (Applied Biosystems). The expression level was determined using cycle threshold (Ct) values, and normalized to mRNA levels of the endogenous control gene peptidyl prolyl isomerase A (*Ppia*). The fold change between the control and experimental groups was calculated using the 2^−ΔΔCT^ formula as previously described (Livak and Schmittgen, 2001). Unless otherwise specified, all chemicals and reagents used were purchased from Sigma-Aldrich (St. Louis, MO, USA). Primers for quantitative PCR were purchased from Integrated DNA Technologies (IDT, San Diego, CA, USA).

**Table 1:** Sequence of the primers used in this study.

Primer name	Primer sequence	Sequence ID
Il-1β-F	ACAGGCAGTGGGAACACTCCT	XM_006529381.2
Il-1β-R	CTCCCCTCTCATCAGCCCTGT	
Mcp-1-F	TTAAAAACCTGGATCGGAACCAA	NM_011333.3
Mcp-1-R	GCATTAGCTTCAGATTTACGGGT	
Gdf15-F	CCGGATACTCAGTCCAGAGG	XM_011242304.2
Gdf15-R	TTCAGGGGCCTAGTGATGTC	
Gadd45β-F	CACCCTGATCCAGTCGTTCT	NM_008655.1
Gadd45β-R	TGACAGTTCGTGACCAGGAG	
Ppia-F	CCATGGCAAATGCTGGACCA	NM_008907.1
Ppia-R	TCCTGGACCCAAAACGCTCC	

### Cochlear Tissue Processing and Immunolabeling

Adult mice were intracardially perfused with 4% paraformaldehyde in 0.01 M phosphate buffer solution (PBS). Right cochleae were harvested and treated for an additional 2 h in 4% paraformaldehyde before decalcification in 10% EDTA for 48 h. Each cochlea was micro-dissected into six pieces, which were later transferred into 30% sucrose for 20 min. Then, non-specific binding sites were blocked by treatment with 5% normal horse serum and 1% Triton X-100 in PBS for 1 h before immunolabeling with primary antibodies. C-terminal binding protein 2 (anti-CtBP2, 1:100; BD Biosciences, San Jose, CA, USA) was used as a primary antibody with overnight incubation, followed by secondary antibody Alexa Fluor 568 (1:500, Invitrogen, Carlsbad, CA, USA). In addition, Alexa Fluor 488 Phalloidin (1:1000, Invitrogen) was applied for visualizing the apical structures of hair cells. Finally, specimens were washed three times and post-fixed with 4% paraformaldehyde for 15 min.

### Confocal Imaging and Data Analysis

Specimens were whole-mounted in VectaShield (Vector Labs, Burlingame, CA, USA) and immunostaining visualized using a Zeiss laser scanning confocal system microscope (LSM 710, Carl Zeiss Microscopy, Thornwood, NY, USA), with a high-resolution oil immersion objective Plan-Apochromat 63× and 1.4 Numerical Aperture. Confocal z-stack images were collected at the cochlear locations equivalent to 16, 24, 32 kHz (Müller et al., 2005). For each set of experiments, all specimens in each group of experimental and control tissues were imaged at the same laser intensity and gain settings.

The count, size and fluorescent intensity of ribbon synapses were measured and quantified using ImageJ (NIH). In brief, z-stacks of each frequency location were first converted to Z-projection and each ribbon particle identified. After ribbon particles were counted manually the numbers were again re-checked with ROI (region of interest) manager, which removed non-specific puncta except for ribbon synaptic particles, and the results then summarized. The measurement of fluorescent particles was quantified by ribbon particle area size, grayscale value and integrated density. Each ImageJ 16-bit image was converted to grayscale, after threshold levels were adjusted to highlight fluorescent ribbon structures, then the binary version of the image was created and designated for the watershed process.

Data were analyzed using Prism (GraphPad Software, La Jolla, CA, USA) software for Windows. The group size *(n)* *in vivo* was determined by the variability of measurements and the magnitude of the differences between groups. Statistical methods used include two-way ANOVA with Bonferroni multiple comparisons, and Student’s unpaired *t*-tests. All tests were two tailed, and a *P* value of <0.05 was considered statistically significant.

## Results

### DARC Is Not Required for the Normal Development of Cochlear Function

**Figure 1:**
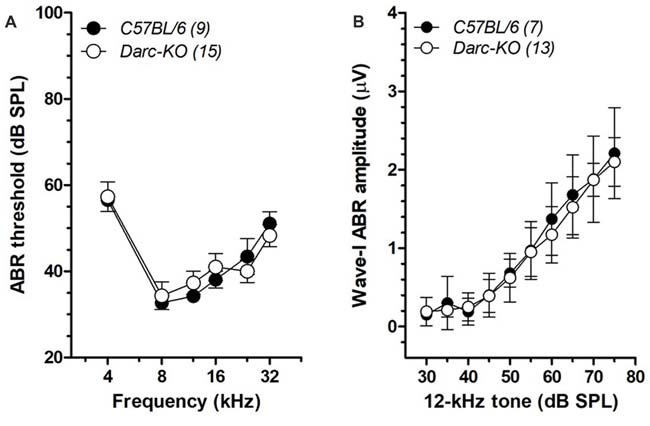
Normal baseline hearing functions in *Duffy antigen receptor for chemokines (DARC)-*KO mice. **(A)** Auditory brainstem response (ABR) thresholds from 4 kHz to 32 kHz were measured in *Darc-*KO (open circle) and age-matched wild type (WT) *C57BL/6* (filled) mice at 6 weeks of age, indicating comparable baseline hearing sensitivity (2-way ANOVA, *P* > 0.05). The number of tested mice is indicated in parentheses. **(B)** The Wave-I peak-to-peak amplitudes of ABR responses to 12-kHz tone pips were extracted, and input/output functions constructed for both *Darc-*KO and age-matched WT *C57BL/6* mice, indicating comparable supra-threshold response (2-way ANOVA, *P* > 0.05).

To evaluate the role of DARC in the cochlear function, baseline hearing sensitivity was first examined in 6-week-old *Darc* deficient mice (*Darc-*KO) and *B6* control mice. No overt difference in ABR thresholds, over the range from 4 kHz to 32 kHz, was observed between the two groups (Figure 1A; 2-way ANOVA, *P* = 0.82). In addition, the ABR Wave-I amplitudes, measured as peak-to-peak voltage difference, were further analyzed in *Darc-*KO mice and input/output functions constructed. No overt difference in ABR supra-threshold responses was observed between the two groups (Figure 1B; 2-way ANOVA, *P* = 0.29), suggesting that deletion of the DARC gene has no effect on ordinary afferent synaptic integrity. Collectively, these data suggest that DARC is not required for the normal development of cochlear function.

### Improved Hearing Recovery After Acoustic Overexposure With Darc Deficiency

**Figure 2:**
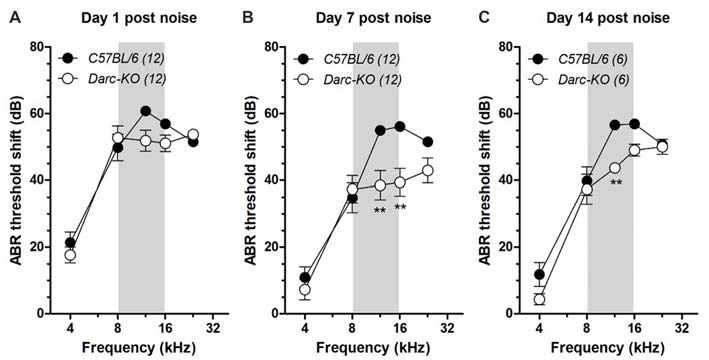
*Darc-*KO mice exhibited improved recovery in hearing sensitivity after noise exposure. Intense octave-band noise (OBN) exposure (112 dB SPL, 8–16 kHz, 2 h, depicted by gray area) caused broad-range elevation of ABR thresholds, observed 1 day, 7 days and 14 days after noise exposure. **(A)** One day after noise exposure, overt ABR threshold shifts were observed in both *Darc-*KO and *C57BL/6* control mice across examined frequencies. Large threshold shifts (>50 dB) were seen at and above the OBN in frequency (i.e., >8 kHz). The number of tested mice is indicated in parentheses. Error bars are SEM. **(B)** Seven days after noise exposure, the ABR threshold shift in *Darc-*KO mice was significantly improved compared to that in WT mice (2-way ANOVA, *P* < 0.0001). *Post hoc* analysis using Bonferroni procedure to correct for multiple comparisons indicated significant (**P < 0.01) *Darc*-dependent hearing recovery at 12 and 16 kHz. **(C)** The improved hearing recovery in *Darc-*KO mice was maintained up to 14 days after noise exposure, examined in another set of animals (2-way ANOVA, *P* = 0.0003). *Post hoc* test of Bonferroni multiple comparisons indicated significant (**P < 0.01) *Darc*-dependent hearing recovery at 12 kHz.

To induce cochlear inflammation, we exposed mice to octave-wide band noise, from 8 kHz to 16 kHz at 112 dB SPL for 2 h (Fujioka et al., 2006; Tan et al., 2016), and examined ABR thresholds at 1 day, 7 days and 14 days after noise exposure (Figure 2). At 1-day post-noise exposure, noise-induced ABR threshold shifts were comparable between the two lines of mice across all frequencies examined (Figure 2A; 2-way ANOVA, *P* = 0.093). Seven days after noise exposure, evidence of recovery of ABR thresholds was across all frequencies in *Darc-*KO mice, while recovery appeared limited in wild-type mice within the frequencies examined, including 4 kHz and 8 kHz, where a 10–15 dB recovery was observed during the weeklong rehabilitation (Figure 2B). Thus, ABR threshold shifts were statistically different in the two lines of mice (2-way ANOVA, *P* < 0.0001). *Post hoc* Bonferroni multiple comparison tests indicated that the ABR thresholds measured in *Darc-*KO mice were significantly lower than *B6* mice at 12 kHz and 16 kHz (*P* < 0.01). Fourteen days post-noise exposure, no further hearing recovery occurred (Figure 2C) in either strain. Although threshold shifts in *Darc-*KO mice were not as deviant from those in *B6* mice, the general threshold shift pattern was equal to that recorded at day 7 post-noise exposure. The deviance was persistent for the ABR threshold shifts between the two lines of mice (*P* < 0.0001). *Post hoc* Bonferroni multiple comparison tests indicated that the ABR thresholds measured in *Darc-*KO mice were significantly lower compared to those measured in *B6* mice at 12 kHz (*P* < 0.01). Collectively, these data indicated *Darc-*KO displayed improved hearing recovery after acoustic overexposure implicated with cochlear inflammation.

### Reduced Inflammation and Increased Anti-inflammatory Factors in Darc-KO Cochleae

**Figure 3:**
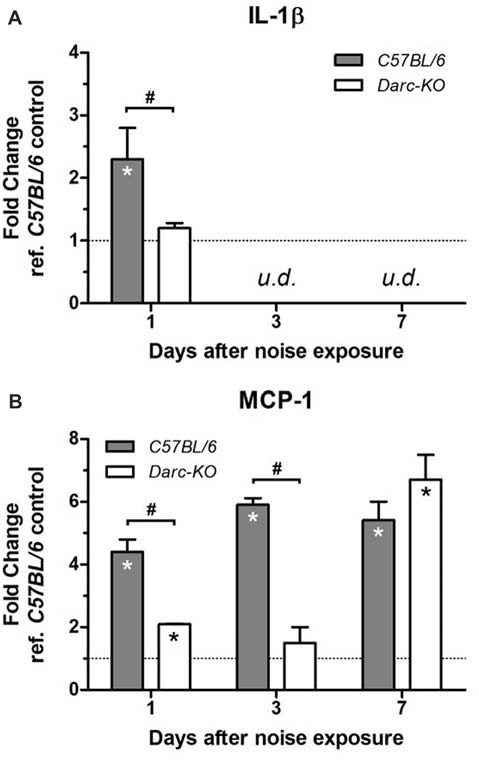
*Darc* deficiency caused a reduction in the mRNA expression of *Il-1β* **(A)** and* Mcp-1* **(B)** in response to acute noise exposure. Mice were exposed to intense OBN (112 dB SPL, 8–16 kHz, 2 h). Then, cochleae were harvested at 1, 3 and 7 days post-noise exposure, and the expression levels of *Il-1β* and *Mcp-1* were evaluated by Real Time PCR in WT and *Darc*-KO mice in comparison with WT control mice not exposed to noise. Data are presented as fold change vs. WT control not exposed to noise, in the format of mean ± SEM. *n* = 3–5, *P > 0.05 vs. control not exposed to noise, ^#^*P* > 0.05 between the two lines of mice exposed to noise. The expression level of *Il-1β* was undetectable (*u.d*.) at 3 and 7 days post-noise exposure.

Changes in the mRNA level of two major inflammatory cytokines in response to noise exposure were evaluated in both WT *B6* and *Darc-*KO mice; interleukin 1 beta (Il-1β) and the DARC-binding monocyte chemotactic protein 1 (MCP-1, *a.k.a*. CCL2). While mRNA levels of *Il-1β* increased as early as 1 day post-noise exposure in cochleae isolated from WT B6 mice (Figure 3A; *P* = 0.03; from here and onward, “WT control” refers to WT *B6* cochleae without noise exposure), mRNA levels of *Il-1β* in *Darc*-KO mice were unaffected by noise exposure (*P* = 0.3). In contrast, Mcp-1 mRNA levels increased in cochleae from both WT and *Darc*-KO as early as 1 day post-noise exposure, but the magnitude of increase was significantly less in cochleae isolated from *Darc-*KO mice as compared to WT B6 mice (*P* < 0.05). Furthermore, *Il-1β* mRNA levels returned to baseline at 3 and 7 days post-noise exposure (Figure 3A, undetectable by our protocol) in both lines of mice, while *Mcp-1* mRNA levels remained significantly elevated as long as day 7 post-noise exposure, in cochleae isolated from WT mice (Figures 3B, *P* < 0.01). However, *Mcp-1* mRNA levels in *Darc-*KO mice did not show any difference (*P* = 0.7) compared to WT control mice at 3-day-post-noise exposure, but by day 7 post-noise exposure (Figure 3B, *P* = 0.004); levels were increased significantly in *Darc-*KO mice compared to WT control mice and became comparable to levels observed in WT mice (Figure 3B). When compared with the noise-induced mRNA expression of *Mcp-1* at multiple time points after noise exposure, 2-way ANOVA indicated the difference in expression between the two lines was statistically significant (*P* = 0.0003).

Expression levels of two other genes, in response to noise exposure, were evaluated to reveal more insights on the pathways that contributed to the alleviated noise-induced ear damage and the expedited recovery phenotype exhibited by *Darc-*KO mice; GDF15, growth differentiation factor, an anti-inflammatory factor and Gadd45β, growth arrest and DNA damage-inducible 45 beta, one of the anti-apoptotic genes that acts through NF-κB activation to suppress pro-apoptotic JNK signaling (De Smaele et al., 2001; Tang et al., 2001; Jin et al., 2002; Papa et al., 2004).

**Figure 4:**
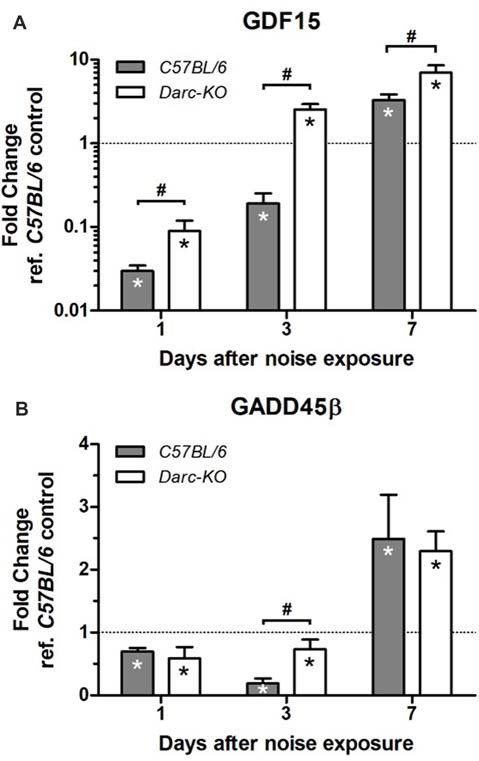
*Darc* deficiency improved the response of *Gdf15* **(A)** and *Gadd45β* **(B)** to noise exposure during the first 7 days post-noise exposure. Mice were exposed to intense OBN (112 dB SPL, 8–16 kHz, 2 h). Then, cochleae were harvested at three time point’s post-noise exposure, and the mRNA expression of *Gdf15* and *Gadd45β* was evaluated by Real Time PCR in WT and *Darc*-KO mice in comparison with WT control not exposed to noise. Data are presented as fold change vs. WT control not exposed to noise, in the format of mean ± SEM. *n* = 4–5, *P > 0.05 vs. WT control not exposed to noise, ^#^*P* > 0.05 between the two lines of mice exposed to noise.

At 1-day post-noise exposure when *Mcp-1* mRNA level was increased significantly in both lines of mice exposed to noise compared to WT control, mRNA levels of both *Gdf15* and *Gadd45β* were significantly reduced in the cochleae of both the C57BL/6 and DARC-KO lines as compared to WT control mice. At day 7 post-noise exposure, mRNA levels significantly increased in both lines compared to WT control mice (Figure 4). At day 3 post-noise exposure, *Gdf15* mRNA levels were reduced in the cochleae isolated from WT mice (0.2-fold, *P* = 0.008) but in *Darc-*KO cochleae, *Gdf15* mRNA levels were significantly increased compared to WT controls (2.5-fold, *P* = 0.03). Furthermore, while *Gadd45β* mRNA levels were reduced significantly at 1 and 3 days post-noise exposure in cochleae of both lines of mice compared to WT control, the effect of noise on *Gadd45β* mRNA levels in WT mice (0.2-fold, *P* = 0.002 vs. WT control) was greater when compared to *Darc-*KO mice (0.7-fold, *P* = 0.05 vs. WT control, *P* < 0.05 between genotypes). Comparing noise-induced mRNA expression of *Gdf15* and *Gadd45β* at multiple time points after noise exposure, 2-way ANOVA indicated significant differential expression between the two lines for *Gdf15* (*P* = 0.002), but not for *Gadd45β* (*P* = 0.80).

In summary, our data are consistent with the hypothesis that a significant reduction in inflammation explains the better prognosis following NIHL in KO mice compared to WT *B6* mice.

### Darc Deficiency Ameliorated Noise-Induced Hair Cell Damage

**Figure 5:**
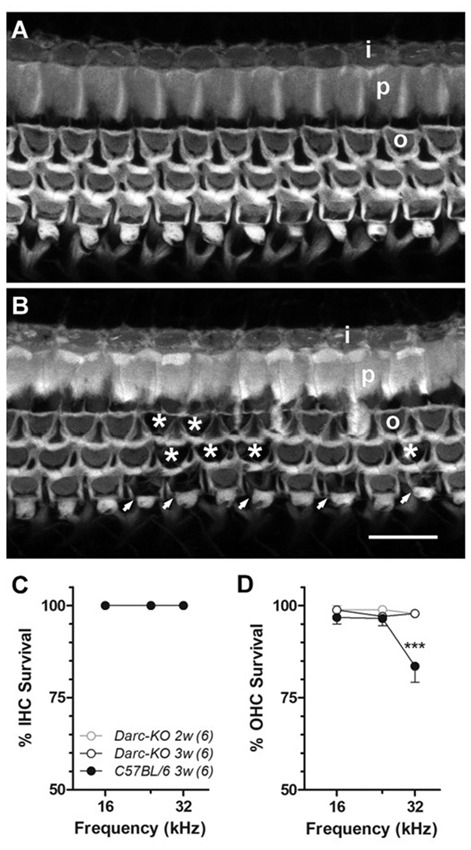
Noise-induced hair cell loss was reduced in *Darc-*KO mice. **(A)** Fluorescently labeled phalloidin revealed well-preserved anatomical integrity of the organ of Corti in *Darc-*KO, 3 weeks post-intense noise exposure, at 32 kHz frequency location. **(B)** Substantial outer hair cell (OHC) (o) loss was observed in the same cochlear location (32 kHz) in WT mice. Missing OHCs were depicted either by asterisks or by arrows. Inner hair cells (IHCs) (i) and pillar cells (p) appeared normal. Scale bar is 10 μm. **(C)** No IHC (I) loss was identified at the examined frequency locations including 16, 24 and 32 kHz, in either *Darc-*KO mice 2- and 3-week after noise damage (*n* = 6, 6), or in control mice 3-weeks after noise (*n* = 6). **(D)** While substantial OHC loss was seen in control mice 3-week after noise exposure, only sporadic OHC loss could be identified in *Darc-*KO mice 2- and 3-week after noise. 2-way ANOVA analysis indicated significantly higher OHC survival overall in *Darc*-KO mice (*P* = 0.003) and *post hoc* Bonferroni multiple comparison tests indicated significantly better OHC survival at 32 kHz in *Darc*-KO mice (***P < 0.001) compared to WT mice. Error bars are SEM.

In addition to noise-induced cochlear inflammation, we have looked for the existence of morphological alterations along the organ of Corti, focusing at the frequencies of 16 kHz, 24 kHz and 32 kHz. This frequency range was above the octave band of noise (OBN) energy and is considered as the most detrimental area in the cochlea. With this particular, intensive noise paradigm, we first qualitatively looked at the hair cell survival by filamentous Phalloidin labeling (Figure 5). At the 32 kHz location, we observed overt noise-induced outer hair cell (OHC) loss in WT mice (Figure 5B), while loss was only sporadic in *Darc-*KO mice (Figure 5A, no cell loss in this particular image). No IHC loss was detected in either of the mouse strains at the three examined frequencies, i.e., 100% IHC survival (Figure 5C). In contrast, OHC loss was detected, but the rate of cell survival was greater in *Darc-*KO cochleae compared to WT cochleae 3 weeks after noise exposure (Figure 5D; 2-way ANOVA, *P* = 0.003). *Post hoc* Bonferroni multiple comparison tests indicated that the OHC survival in *Darc-*KO mice were significantly better than WT mice at 32 kHz (*P* < 0.001).

### Darc Deficiency Mitigated Noise-Induced Cochlear Synaptopathy

**Figure 6:**
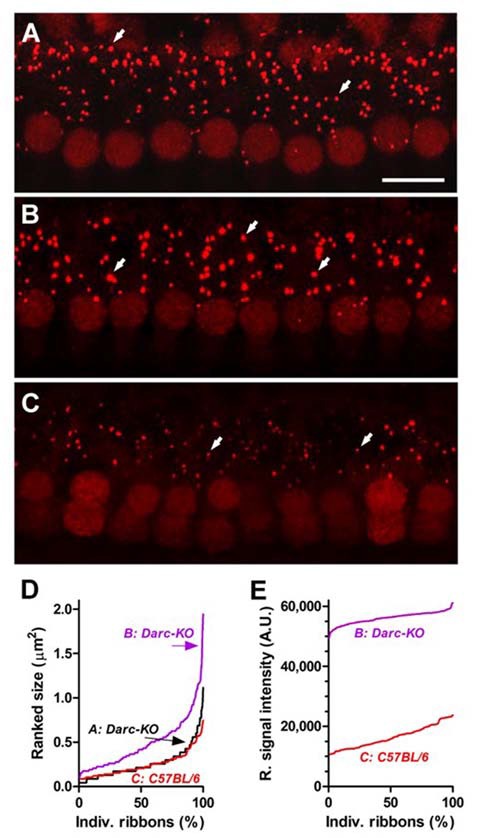
*Darc* deficiency eased the noise-induced damage on synaptic ribbons of IHCs at cochlear location equivalent to 24 kHz. **(A)** Fluorescent anti-CtBP2-labeled particles (arrows), identified presynaptic ribbons along the basolateral membrane of the IHCs from a representative z-project confocal image acquired from an un-treated *Darc-*KO cochlea at 24 kHz frequency location. Arrows depict individual ribbons. Scale bar is 10 μm. **(B)** Three weeks after noise exposure (OBN, 112 dB SPL, 8–16 kHz, 2 h), robust fluorescent anti-CtBP2-labeled particles could be easily identified from a *Darc-*KO cochlea at 24 kHz frequency location. **(C)** In comparison, the fluorescent particles in a representative noise-exposed WT *C57BL/6* cochlea displayed lower fluorescent signal strength and smaller particle size. Ranked particle size **(D)** and ranked signal intensity **(E)** from individual fluorescent particles are graphed for quantitative comparison.

**Figure 7:**
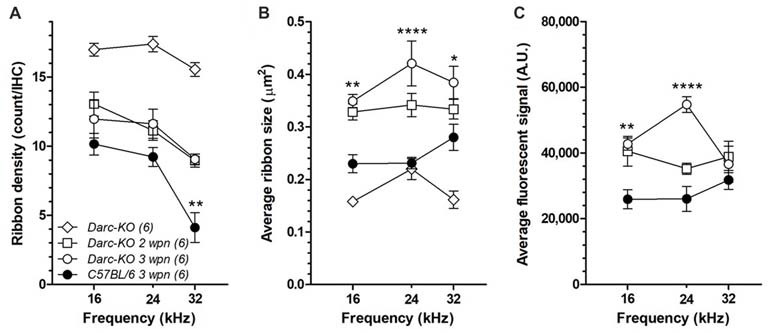
*Darc* deficiency reduced the noise-induced damage on synaptic ribbons of IHCs at the frequency region above the OBN. **(A)** Without noise damage (open diamond), the baseline density of ribbon synapse from *Darc-*KO mice (*n* = 6) was comparable to healthy WT *B6* mouse cochleae (Wan et al., 2014), ranging from 15 to 18 ribbons per IHC. Noise damage reduced the number of presynaptic ribbons in both *Darc-*KO and control WT *B6* cochleae (*n* = 6, 6), examined at frequency locations including 16, 24 and 32 kHz. However, the number of survived presynaptic ribbons per IHC was significantly higher in *Darc-*KO mice (2-way ANOVA, *P* < 0.0001). Average particle size **(B)** and average signal intensity **(C)** were also calculated from frequency locations including 16, 24 and 32 kHz, in both *Darc-*KO mice 2- and 3-week after noise damage (*n* = 6, 6), and in WT mice 3-weeks after noise (*n* = 6). Three weeks after noise, particle size was larger and ribbon signal higher in *Darc-*KO cochleae compared to controls (2-way ANOVA, *P* < 0.0001). Error bars are SEM. A.U. = arbitrary unit. Asterisks indicate the significance of corresponding *post hoc* Bonferroni multiple comparison tests. (*P < 0.05, **P < 0.01, ****P < 0.0001).

Since DARC is known to play a significant role in inflammation, in this study, we made use of the *Darc*-KO mouse model to determine if DARC is involved in presynaptic ribbon loss after traumatic noise exposure. In untreated *Darc*-KO mice, baseline density of ribbon synapses ranged from 15–18 ribbons per IHC across three examined locations (Figure 7A), comparable to previously published healthy WT *B6* mouse cochleae (Wan et al., 2014). The count of CtBP2-labeled presynaptic ribbons was 16.2 ribbons per IHC at the cochlear location equivalent to 24 kHz from a representative fluorescent image (Figure 6A). Following intensive noise exposure, we observed loss of CtBP2-labeled presynaptic ribbons in WT *B6* cochlea examined 3 weeks after noise exposure (Figures 6C, 7A). In a representative image ribbon density decreased to 6.6 ribbons per IHC at 24 kHz location (Figure 6C). As the size and fluorescent signal strength of individual synaptic ribbon particles varied greatly, it was challenging to identify smaller ribbons with weak fluorescent signals (Figures 6D,E). In contrast, large synaptic ribbons and robust fluorescent signals were observed in organs of Corti taken from *Darc-*KO cochleae (Figures 6B,D,E). A representative image shows a ribbon density of 11.0 ribbons per IHC at 24 kHz location 3 weeks after noise exposure (Figure 6B). Group data showed very similarly reduced ribbon density in KO cochleae at 2 weeks and 3 weeks after noise exposure (Figure 7A), although the density was significantly greater than that observed in WT mice (Figure 7A; 2-way ANOVA, *P* = 0.0004). Statistical analysis on group data also confirmed the trend of the outperformance observed in *Darc-*KO mice after acoustic overexposure for both synapse size and fluorescence signal intensity (Figures 7B,C). Two-way ANOVA indicated significantly larger synaptic ribbons (Figure 7B; *P* < 0.0001), marked by robust fluorescence signals (Figure 7C; *P* < 0.0001). *Post hoc* Bonferroni multiple comparison tests indicated that these attributes in *Darc-*KO mice were in general greater than in WT *B6* mice at the three individually surveyed frequencies, depicted by the asterisks in the figure panels (*P < 0.05, **P < 0.01, ****P < 0.0001). Baseline synaptic ribbons from *Darc*-KO mice were assessed at equivalent ages to when noise exposure was conducted, and mice sacrificed 3 weeks later for age-matched comparison. Note that the images from baseline *Darc*-KO cochleae were collected at a different time with altered equipment settings, so that comparison of ribbon particle fluorescence strength was not possible. However, we were able to manually count baseline ribbon numbers (Figure 7A) and measure size using ImageJ (Figure 7B).

It is noteworthy to consider the differential acoustic damage of the *Darc*-dependent noise-induced hair cell loss and synaptic ribbon loss. In the present study, the *Darc*-dependent noise-induced cochlear inflammation resulted in both OHC loss and synaptic ribbon deficit. The hair cell loss was identified by filamentous phalloidin labeling, and was limited only to OHCs at higher frequency regions, i.e., 32 kHz (Figure 5), while synaptic deficit was observed at all three surveyed frequencies examined jointly by ribbon count, size and fluorescent signal strength (Figure 6). This differential damage collectively suggests that cochlear inflammation is probably more detrimental to neuronal synaptic terminals, than it is to the filamentous structure of hair cells.

## Discussion

Cytokines and chemokines are produced by a variety of cell types and are known to regulate cell recruitment and migration in response to inflammation during wound healing. Recent studies have suggested that inflammation plays an important role in the pathology of hearing loss induced by intense noise exposure (Tan et al., 2016; Fuentes-Santamaria et al., 2017). However, the roles of chemokines and their receptors in NIHL are not yet well established.

DARC, binds pro-inflammatory chemokines such as MCP-1 (Szabo et al., 1995), and is expressed in cells known for their role in inflammation and wound healing. Data from our previous studies have revealed the importance of DARC in response to bacterial infection and post fracture inflammation (Rundle et al., 2013; Alemi et al., 2016). However, the role of DARC in NIHL has never been investigated. Therefore, the goal of this study was to determine whether a loss of the DARC gene had any effect on noise-induced inflammation, hearing loss and/or recovery.

### Alteration of Noise-Induced Cochlear Inflammation

It is now well accepted that inflammation plays a significant role in NIHL (Fujioka et al., 2006; Tan et al., 2016). In the present study, exposure to intense noise induced the expression of *Il-1β*, a major pro-inflammatory cytokine, and MCP-1, one of the key inflammatory chemokines that binds to DARC. At the same time, exposure to intense noise reduced the expression of the anti-inflammatory effectors GDF15 and Gadd45β. This produced a broad range elevation of ABR thresholds confirming the involvement of inflammation in NIHL. The effect of noise exposure on expression of the pro/anti-inflammatory cytokines tested in this study was significantly less in KO mice compared to WT mice. Lack of *Darc* expression in KO mice attenuated the response of Il-1β to acute noise exposure as early as 1-day post-noise exposure, and altered the response to noise exposure of one of the key chemokines that regulate inflammation.

Levels of GDF15 mRNA, a cytokine involved in regulating inflammatory and apoptotic pathways, have been shown to increase following cardiac injury (Ago and Sadoshima, 2006; Kempf et al., 2011), the presumption being that this protects cardiac cells from apoptosis. In addition, it has been reported that GDF15 reduces leukocyte recruitment by modulating chemokine signaling (Kempf et al., 2011). In our study, the alteration in the expression of GDF15 caused by acoustic trauma recovered earlier in cochleae isolated from *Darc*-KO mice when compared to WT mice (Figure 4). This suggests that a lack of DARC results in early resolution of noise-induced inflammation, which in turn results in protection from noise induced hair loss and synaptic damage, as evidenced by the greater density of the presynaptic IHC ribbon of *Darc*-KO mice compared to WT mice post-noise exposure (Figure 6).

The effect of *Darc* deficiency on mRNA levels of all the genes tested in this study occurred relatively early following noise exposure. However, at day 7 following exposure, only the expression of *Gdf15* remained significantly elevated in KO mice as compared to WT mice (by both *t*-test and *Post hoc* Bonferroni multiple comparison test), while expression of *Mcp-1* and *Gadd45β* was equally increased in the two lines of mice. Previous studies (Tan et al., 2016) have reported an early induction in the expression of *Mcp-1* followed by another peak at day 7 following noise exposure, but in our study, the mRNA level of *Mcp-1* showed two peaks only in cochleae isolated from KO mice, while it remained greater than no-noise control during all 7 days post-noise exposure in WT mice.

The occurrence of the latter expression peak at day 7 following noise exposure has been reported previously (Tan et al., 2016), but the significance of its occurrence has never been investigated. MCP-1 is known to play a significant role in inflammation, and it is a key attractant of monocytes and macrophages. However, it has been reported that MCP-1 can decrease hypoxia-induced cell death in cultured cardiac monocytes (Tarzami et al., 2002), and it is necessary for both the anabolic and catabolic effects of parathyroid hormone on bone cells (Siddiqui and Partridge, 2017). Therefore, we hypothesize that the first peak in *Mcp-1* mRNA expression was due to inflammation induced by noise exposure, while the latter peak was associated with the reparative process in KO mice. In WT mice, the levels of *Mcp*-1 mRNA remained elevated in mice exposed to intense noise compared to controls during the 7 days post-noise exposure period. This suggests that prolonged inflammation in WT mice following noise exposure leads to irreversible hair cell and synaptic damage.

Our findings show for the first time that *Darc* deficiency in KO mice provides protection from noise-induced hair loss and neuronal damage, in part through changes in MCP-1 catabolic and anabolic functions.

### Reduced Risk of Hearing Loss in Africa Americans, the Implication of Darc Deficiency

In humans, the Duffy blood group system consists of four alleles, five phenotypes and five antigens (Pogo and Chaudhuri, 1995, 2000). Duffy-negative individuals are predominantly of African origin. They have been shown to lack the Duffy protein on erythrocytes and are more resistant to *Plasmodium vivax* and *Plasmodium knowlesi* infections than Duffy positive individuals (Miller et al., 1975, 1976). Intriguingly, it has been long known that African-Americans exhibit significantly less age-related hearing loss when compared to people of other ethnic backgrounds (Jerger et al., 1986; Helzner et al., 2005; Lin et al., 2012). After adjustment for age, African Americans also exhibit significantly better resistance to NIHL, in studies involving cross-sectional samples of metal fabricating workers (Ishii and Talbott, 1998) and gold miners (Strauss et al., 2014). Melanin levels in cochlear melanocytes have been speculatively reasoned as the source of the race/ethnicity differences in the variation of susceptibility to NIHL, and there has been substantial research performed both on animal models (Hayashi et al., 2007; Ohlemiller et al., 2009; Murillo-Cuesta et al., 2010), and using human temporal bones (Sun et al., 2014). Our finding of resistance against NIHL and noise-induced cochlear damage in *Darc-*KO mice compared with WT mice raises the interesting possibility that variation of susceptibility to NIHL between African-Americans and Caucasians could be due in part to a difference in the expression of *Darc* between the two groups.

### Darc-Dependent Amelioration of Noise-Induced Cochlear Damage May Also Be Due to Enhanced Cochlear Angiogenesis

Increased cochlear vascular endothelial growth factor (VEGF) expression was observed in noise-exposed animals, particularly at the level of the *stria* *vascularis*, spiral ligament and spiral ganglion cells (Picciotti et al., 2006). VEGF is a prototypical angiogenic factor that exerts direct effects on neural cells and several other cells. Abnormal regulation of VEGF expression has been implicated in neurodegenerative disorders (Storkebaum and Carmeliet, 2004). Whether VEGF is differentially expressed in *Darc-*KO mice after noise exposure was beyond the scope of this study. However, a previous study observed that the expression of DARC on endothelial cells had attenuating effect on vascular capillary growth (Xu et al., 2007). We cannot rule out that *Darc* deficiency in our mouse model also enhances angiogenesis, which would be clinically significant in diseases like peripheral and coronary ischemia and infarction, chronic wound healing failure and ulcers, all being characterized by insufficient angiogenesis. It is also known that angiogenesis, neurogenesis and synaptic plasticity naturally occur in the neural system upon neural repair (Font et al., 2010). The observation of robust *Darc*-dependent fluorescent signals present in the synaptic ribbon supports a role of DARC during the process of neurorepair. Thus, developing a drug to block or attenuate the activity of DARC might enhance angiogenesis and neurogenesis, in addition to regulate neuroinflammation, and subsequently protect cochlear function central to hearing.

### Inflammation-Dependent Cochlear Damage Is Implicated in Cochlear Synaptopathy

In the present study, we compared the morphological deterioration of cochlear hair cells and the ribbon synaptic deficit at the synapses of the IHC and afferent dendrites. Our results indicate that cochlear inflammation is more detrimental to neuronal synaptic terminals, than it is to the hair cell filamentous structure. Modulating the level of inflammation might allow for complete survival of hair cells and selective damage in the ribbon synapse, i.e., the characteristic cochlear synaptopathy or “hidden” hearing loss (Kujawa and Liberman, 2009, 2015; Liberman and Kujawa, 2017). Thus, this study supports the hypothesis that cochlear inflammation is one of the underlying mechanisms that contribute to synaptopathy. In addition, cochlear inflammation can be derived by many innate or environmental factors, such as the exposure to noise used in this study. Other factors including but not limited to autoimmune inner ear diseases, and traumatic brain injuries, might be expected to result in cochlear inflammation as well. We speculate that regardless of the cause of cochlear inflammation, we would expect to observe an elevated risk of cochlear synaptic deficit, manifested by poor temporal processing ability and difficulty listening in a noisy environment.

As DARC deficiency improved hearing recovery following acute noise exposure, larger synaptic ribbons and robust fluorescent signals were evident in the inflammation suppressed model (Figures 6B,D,E). The observation that ribbon enlargement is associated with functional recovery from noise trauma is reminiscent of a similar phenomenon that occurs during neonatal cochlear development. In that case a size increment in ribbon synapses occurs between postnatal day 3 and day 10, followed by size reduction by 3 weeks (Kalluri and Monges-Hernandez, 2017). The structure of the ribbon itself is suggestive that it plays an important role in both synaptogenesis and synaptic regeneration. In adult animals, large ribbons are more abundantly distributed in the modiolar and basolateral zone. They are innervated primarily by auditory nerve fibers having a low-medium spontaneous rate, which are acoustically responsible for conveying supra-threshold cues (Liberman et al., 1990, 2011; Merchan-Perez and Liberman, 1996). Thus, the ribbon enlargement observed in Darc-KO mice may also implicate a protective mechanism that preferentially benefits supra-threshold hearing during hearing recovery.

As we now know that the inner ear is not completely “immune privileged,” immune activities, including the inflammation process, have been retained in the mammalian cochlea. Inflammatory modulators such as DARC provide important functions across the blood-labyrinth barrier. Further investigation is required to better understand the long-term effect that *Darc*-deficiency might have under situations that cause cochlear inflammation, such as acoustic overexposure. In addition, it will also be necessary to quantify the degree of inflammation-dependent hearing damage that contributes to the overall hearing deficits caused by noise exposure, ototoxic compounds or during the aging process.

In summary, the data from this study show for the first time that DARC plays a significant role in the noise-induced inflammation that leads to synaptic damage and hearing loss. Together, these data, with the alteration of inflammatory cytokine levels in *Darc-*KO mice reported from earlier studies (Rundle et al., 2013; Alemi et al., 2016) demonstrate the importance of DARC in inflammatory responses that lead to pathological states such as hearing loss.

## Author Contributions

BE and HL conceived and designed the project; wrote and revised the article. BE, LS and AH performed the experiments. BE, LS, AH and HL all analyzed the data.

## Conflict of Interest Statement

The authors declare that the research was conducted in the absence of any commercial or financial relationships that could be construed as a potential conflict of interest.
